# Effects of Strip Width on Inter-Row Heterogeneity in Light Interception and Utilization of Intercropped Soybeans

**DOI:** 10.3390/plants15020182

**Published:** 2026-01-07

**Authors:** Yue Li, Yao Zhang, Jiamiao Shi, Ruizhe Zhang, Lisha Zhang, Yuan Yang, Haichang Li, Lihua Wang, Tianyu Yuan, Sirong Huang, Xiaochun Wang, Feng Yang, Jiang Liu, Taiwen Yong, Yanhong Yan, Wenyu Yang, Yushan Wu

**Affiliations:** 1 College of Agronomy, Sichuan Agricultural University, Chengdu 611130, China; 2 Sichuan Engineering Research Center for Crop Strip Intercropping System, Key Laboratory of Crop Eco-Physiology and Farming System in Southwest, Chengdu 611130, China; 3 College of Life Science, Sichuan Agricultural University, Yaan 625014, China; 4 College of Grassland Science and Technology, Sichuan Agricultural University, Chengdu 611130, China

**Keywords:** strip intercropping, strip width, soybean, radiation use efficiency, yield

## Abstract

Strip intercropping improves productivity through enhanced light interception. In this study, we quantified the effects of strip width on light interception of soybean across six strip widths (2.2 m, 2.5 m, 2.8 m, 3.1 m, 3.4 m, 3.7 m) when intercropped with maize. Results showed that photosynthetically active radiation (PAR) in western rows of intercropped soybeans peaked at 11:30 a.m., whereas in eastern rows, it occurred at 1:00 p.m. Across 2.2 m to 3.7 m, PAR in the western rows of intercropped soybeans was 6.1% higher than that of the eastern rows for the whole growth period. During the R5 stage, compared to eastern rows, radiation use efficiency (RUE), dry matter accumulation, and leaf area of soybean in western rows increased by 4.0%, 7.4%, and 6.7%, respectively. Compared to the 2.2 m strip width, grain yields in eastern rows of 2.5–3.7 m strip widths were 8.5%, 54.7%, 56.5%, 63.4%, and 69.0% higher than those of the 2.2 m strip width, respectively. PAR had the strongest influence on dry matter and leaf area at a 3.7 m strip width, while RUE had the strongest influence at 3.1 m strip widths. These findings advance our understanding of light partitioning in strip intercropping and support future climate-adaptive intercropping systems’ modeling.

## 1. Introduction

Intercropping is considered an important planting pattern in modern agriculture for improving farmland utilization and enhancing productivity [1,2]. Traditional row or mixed intercropping is predominantly adopted under low-input and resource-limited conditions, which cannot meet the growing global food demand [3], and lacks supporting management practices, such as synergistic fertilization and weed control for both crops. These shortcomings result in low production efficiency and economic benefits. Strip intercropping could combine the strengths of modern intensification and traditional crop diversification that not only increases resource use efficiency (e.g., water, light, fertilizer, etc.) and achieves high yield but also reduces environmental impacts while maintaining high sustainability [4,5,6]. The improvement in crop complementarity under temporal and spatial conditions is the main reason for yield increase in strip intercropping systems [1,7,8,9,10,11]. Meanwhile, as strip width becomes wider, it is easier to manage fieldwork with existing machinery and meet the demands of modern agriculture [12,13,14,15].

Soybean–maize strip intercropping is an innovation of traditional row intercropping, which combines tall-stature maize (C_4_) and short-stature soybean (C_3_) and plants them in alternating strips at a certain ratio to improve the light energy utilization of crop communities and land productivity. This is an important cropping pattern for maintaining maize production capacity and increasing soybean production [16,17]. The system has been widely promoted in China due to its high productivity and sustainability [15,18], and since 2022, its cumulative promoted area has exceeded 3.73 million hectares across 18 provinces. In this system, alternate strips of different crops lead to canopy heterogeneity and spatial niche differentiation [19], which alters the microclimate, especially light distribution [10,11,20]. Generally, the strongest competitive and facilitative interactions between crops occur in border rows, primarily driven by the capture of key resources (e.g., radiation, water, and nutrients) [7,11,21]. For tall-stature maize, this intercropping pattern enables higher light interception than monocropped maize, particularly in the middle and lower canopy leaves of border rows [22]. In contrast, for short-stature soybean, intercropping limits its light capture and utilization [16,20], especially in the side row that is close to the maize side [21,23]. This limitation is regulated by the strip width [24,25,26]. Competition for aboveground light resources drives adaptive morphological plasticity in soybeans [23], such as decreased stem diameter, specific leaf area, and branching frequency, as well as the inhibition of soybean growth and yield formation [27,28]. Strip width plays an important regulatory role in the environmental–physiological response of soybeans; wider strips could increase soybean pods, seeds, and grain yield but lead to inter-row heterogeneity [6]. For example, limited shading of soybeans by maize planted in the north–south row orientation did not affect the overall yield advantage, but light interception differences between morning and evening caused yield differences between the eastern and western rows [29,30,31]. Thus, quantifying the effects of strip width on inter-row differences in light interception and utilization, and clarifying their contribution to grain yield formation, is necessary to improve light use efficiency and yield of short-stature crops in strip intercropping.

Some studies could be used to summarize the relationship between strip width and light interception of short crops in intercropping species, but most of them could only quantify the overall canopy effect of short-stature crops and ignore inter-row differences [11]. Most previous reports have studied the effect of strip width on the phenotype and physiology of short-stature crops in intercropping. However, the range of strip width they established was not wide enough and failed to identify the optimal strip width for light use efficiency. Modeling is a cost-effective method for quantifying these light-related effects. For instance, simple geometric models based on Beer’s law (e.g., rectangles, ellipsoids, or cylinders) are a common approach [32]; however, this approach overlooks the dynamics of light interception in intercropping systems [33]. Three-dimensional plant models are another method to quantify light interception via geometric modeling, but they require much plant morphological data as input (e.g., leaf pinch angle, number of leaves, and height of the plant), a process that makes data collection time- and labor-consuming [34]. In our previous study, we developed a model to quantify the daily light interception of soybean plants under different strip widths of soybean–maize strip intercropping and verified its accuracy using measured photosynthetically active radiation (PAR) and phenotypic responses [6]; however, this model cannot quantify the light interception throughout the entire growth period of intercropped soybeans, and our strip width range is not sufficiently wide enough. Furthermore, existing light models for strip intercropping do not sufficiently consider all factors that affect light distribution (e.g., latitude, row orientation, etc.) and require further improvement in the prediction accuracy. Monitoring more specific PAR data in the spatiotemporal domain for strip intercropping and quantifying the inter-row heterogeneity of light interception are still necessary for assessing the strip width effects of intercropping, which also provides support for the development of a light interception model for intercropping systems.

In this study, we hypothesized that strip width would affect the light interception of soybeans and subsequently influence their growth and yield formation and their existing inter-row heterogeneity among different soybean rows. We conducted an experiment with six strip widths in soybean–maize intercropping. Our objectives were (1) to quantify the spatiotemporal heterogeneity in photosynthetically active radiation (PAR) of intercropped soybeans across rows under six strip widths; (2) to investigate the inter-row variability of leaf area, biomass, radiation use efficiency (RUE), and grain yield of intercropped soybeans; and (3) to quantify the relationship between PAR and related physiological responses of intercropped soybeans.

## 2. Materials and Methods

### 2.1. Experimental Site

**Figure 1:**
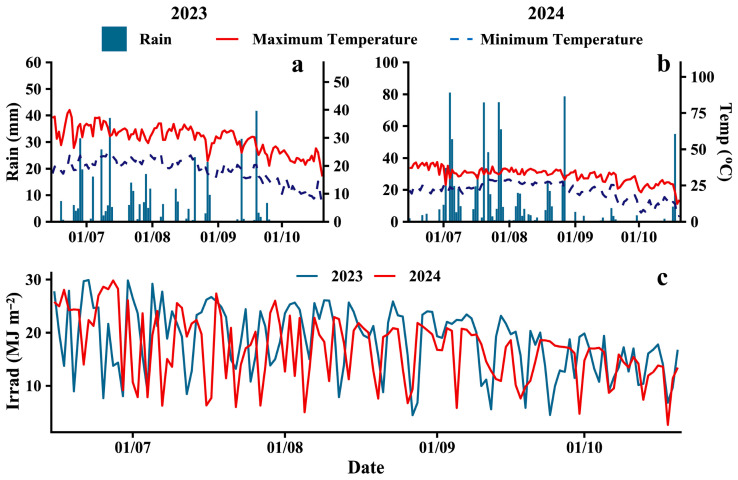
Meteorological data during the soybean-maize intercropping seasons of 2023 and 2024. Air temperature (**a**,**b**) (right), rainfall (**a**,**b**) (left), and solar irradiation (**c**).

A two-year field experiment was conducted from 2023 to 2024 at the experimental farm of Peking University’s Modern Agriculture Research Institute, located in Weifang, Shandong Province, China (36°42′23.9″ N, 119°7′36.6″ E). The site is characterized by a warm-temperate monsoon semi-humid climate, with an annual average temperature of 13.8 °C, a frost-free period ranging from 191 to 213 days, a sunshine duration of 2284.8 h, and an annual precipitation of 856.4 mm. During 2023 and 2024, precipitation was 448.1 mm and 948.4 mm, respectively, with average temperatures of 25.4 °C and 24.3 °C (Figure 1). The soil at the experimental site was sandy loam (soil particle size distribution ranged from 0.002 to 2 mm, bulk density 1.46) according to IUSS Working Group 2014 and 2022, with a pH of 7.68, an organic matter content of 13.48 g kg^−1^, a total nitrogen content of 0.77 g kg^−1^, an available *P* content of 47.79 mg kg^−1^, and an available *K* content of 138.52 mg kg^−1^ in the top 0–40 cm soil layer.

### 2.2. Experimental Design

**Figure 2:**
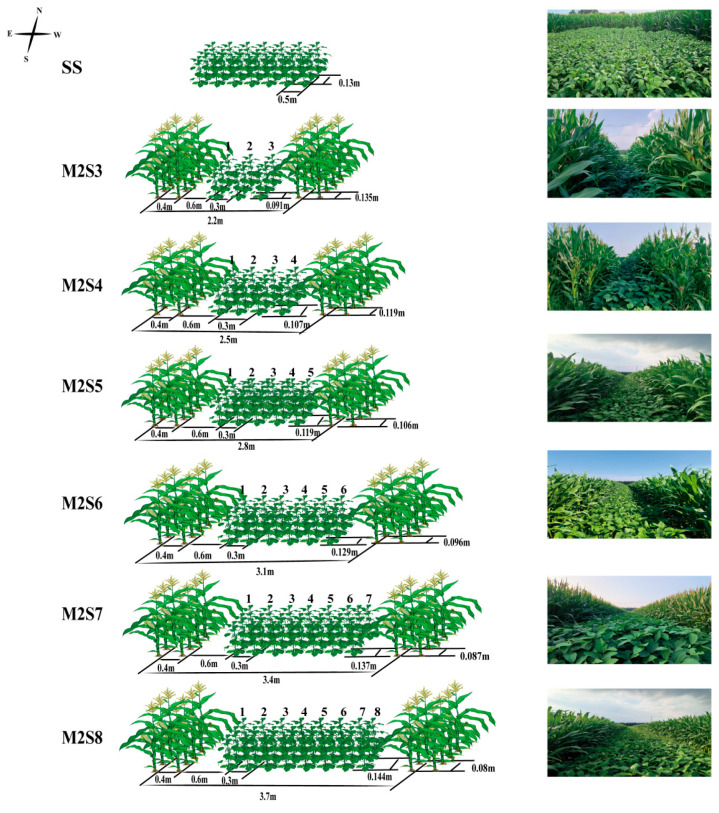
Layout and photos of the experiment in this study from 2023 to 2024. For treatments with an even number of soybean rows, the center of the two central rows was aligned with the center of the soybean strip. For treatments with an odd number of rows, the central row was aligned with the center of the soybean strip. Rows 1 to 8 represent east to west.

The field experiment was laid out in a randomized complete block design with three replicates for each treatment. There were six strip widths of soybean–maize strip intercropping and one sole soybean (SS) treatment as the control (Figure 2). The strip intercropping treatments consisted of two maize rows intercropped with 3–8 rows of soybean: (1) M2S3, 2.2 m strip width; (2) M2S4, 2.5 m strip width; (3) M2S5, 2.8 m strip width; (4) M2S6, 3.1 m strip width; (5) M2S7, 3.4 m strip width; and (6) M2S8, 3.7 m strip width. The sole soybean (SS) was planted with a row spacing of 0.5 m. All treatment plots were 8 m in length. The detailed crop layout is shown in Figure 2.

During the 2023 and 2024 growing periods, direct seeding was conducted on 15 June, and harvesting took place on 15 October. The maize cultivar was Zhengdan 958, and the soybean cultivar was Qihuang 34. Both crops were sown in hills (2–3 seeds per hill) and subsequently thinned to retain one plant per hill. In all treatments the crops were planted in a north–south direction, with a density of 150,000 plants per hectare for both intercropped and sole soybeans and 75,000 plants per hectare for intercropped and sole maize. Maize plants in all treatments received 270 kg N, 54 kg P, and 108 kg K per hectare, whereas soybeans remained unfertilized. We employed drip irrigation, with each drip tube equipped with a bypass valve to regulate water flow. Weed control was performed manually. Pests and diseases were managed through drone-applied pesticide spraying.

### 2.3. Sampling and Measurements

#### 2.3.1. Sampling Time

Sampling was conducted at four key growth stages of soybeans in both 2023 and 2024: the five-leaf stage (V5), pod initiation stage (R3), grain filling stage (R5), and maturity stage (R8).

#### 2.3.2. Leaf Area and Dry Matter

In each experimental plot, three uniformly growing soybean plants were randomly selected from each row for physiological parameter measurements. Initially, all leaves of the selected plants were scanned using a high-resolution scanner, and subsequently, ImageJ v.1.5.4g software was used to calculate the individual plant leaf areas. For dry matter determination, the harvested soybean plants were divided into different organs and subjected to heat treatment at 105 °C for 30 min, followed by drying at 80 °C for 72 h until a constant weight was reached.

#### 2.3.3. Photosynthetic Active Radiation (PAR)

In both 2023 and 2024, PAR data recording began on 20 July, when soybeans were at the V5 growth stage. HOBO sensors (Onest, UA-002-08, Bourne, MA, USA) were utilized to measure the photosynthetically active radiation (PAR) intercepted by the soybean canopy under different treatments. As detailed in Figure S1, the sensors were positioned 10 cm above the canopy of each soybean row for each treatment in the field. The HOBO sensors were set to record PAR values at 15 min intervals.

#### 2.3.4. Yield

At maturity, one-meter sections of each row were excluded from both ends of each plot to minimize edge effects. Then, twenty consecutive maize or soybean plants were randomly selected from each row within the remaining areas. We then measured the number of pods, grains, and 100-seed weight.

### 2.4. Light Interception Fraction

The fractions of canopy light interception for strip intercropping soybean were calculated by the RCRT model (extended row crop radiation transmission), which divided the intercepted PAR into nine parts, and the soybean intercepted fraction was the sum of the parts 6–8. The equations were [35,36]

(1) $$F1=f_{a}\times {\mathrm{Fia}}_{\mathrm{black}}(1-e^{-\frac{k_{a}\times {\mathrm{LAI}}_{\mathrm{ia}}}{f_{a}}})$$

(2) $$F2=f_{a}\times {\mathrm{Fia}}_{\mathrm{black}}(1-e^{-k_{a}\times {\mathrm{LAI}}_{\mathrm{ia}}})$$

(3) $$F3=f_{b}(1-{\mathrm{Fib}}_{\mathrm{black}})(1-e^{-k_{a}\times {\mathrm{LAI}}_{\mathrm{ia}}})$$

(4) $$F4=\mathrm{Fia}\times {\mathrm{Fiia}}_{\mathrm{black}}(1-e^{-\frac{k_{a}\times {\mathrm{LAI}}_{\mathrm{iia}}}{f_{a}}})$$

(5) $$F5=\mathrm{Fia}(1-{\mathrm{Fia}}_{\mathrm{black}})\times (1-e^{-(k_{a}\times {\mathrm{LAI}}_{\mathrm{iia}}+k_{b}\times {\mathrm{LAI}}_{b})})\times \frac{k_{a}\times {\mathrm{LAI}}_{\mathrm{iia}}}{k_{a}\times {\mathrm{LAI}}_{\mathrm{iia}}+k_{b}\times {\mathrm{LAI}}_{b}}$$

(6) $$F6=\mathrm{Fia}(1-{\mathrm{Fia}}_{\mathrm{black}})\times (1-e^{-(k_{a}\times {\mathrm{LAI}}_{\mathrm{iia}}+k_{b}\times {\mathrm{LAI}}_{a})})\times \frac{k_{b}\times {\mathrm{LAI}}_{b}}{k_{a}\times {\mathrm{LAI}}_{\mathrm{iia}}+k_{b}\times {\mathrm{LAI}}_{b}}$$

(7) $$F7=\mathrm{Fia}\times {\mathrm{Fiib}}_{\mathrm{black}}(1-e^{-\frac{k_{b}\times {\mathrm{LAI}}_{b}}{f_{b}}})$$

(8) $$F8=\mathrm{Fib}(1-{\mathrm{Fib}}_{\mathrm{black}})\times (1-e^{-(k_{a}\times {\mathrm{LAI}}_{\mathrm{iia}}+k_{b}\times {\mathrm{LAI}}_{a})})\times \frac{k_{b}\times {\mathrm{LAI}}_{b}}{k_{a}\times {\mathrm{LAI}}_{\mathrm{iia}}+k_{b}\times {\mathrm{LAI}}_{b}}$$

(9) $$F9=\mathrm{Fib}(1-{\mathrm{Fib}}_{\mathrm{black}})\times (1-e^{-(k_{a}\times {\mathrm{LAI}}_{\mathrm{iia}}+k_{b}\times {\mathrm{LAI}}_{a})})\times \frac{k_{b}\times {\mathrm{LAI}}_{\mathrm{iia}}}{k_{a}\times {\mathrm{LAI}}_{\mathrm{iia}}+k_{b}\times {\mathrm{LAI}}_{b}}$$

(10) $$\mathrm{Fia}=f_{a}[{\mathrm{Fia}}_{\mathrm{black}}\times e^{-k_{a}\times {\mathrm{LAI}}_{\mathrm{ia}}}]+f_{a}\times (1-{\mathrm{Fia}}_{\mathrm{black}})e^{-k_{a}\times {\mathrm{LAI}}_{\mathrm{ia}}}+f_{a}f_{b}(1-{\mathrm{Fia}}_{\mathrm{black}})\times e^{-k_{a}\times {\mathrm{LAI}}_{\mathrm{ia}}}$$

(11) $$\mathrm{Fib}=f_{a}[{\mathrm{Fib}}_{\mathrm{black}}+f_{b}\times (1-{\mathrm{Fib}}_{\mathrm{black}})e^{-k_{a}\times {\mathrm{LAI}}_{\mathrm{ia}}}]+f_{a}f_{b}(1-{\mathrm{Fia}}_{\mathrm{black}})\times e^{-k_{a}\times {\mathrm{LAI}}_{\mathrm{ia}}}$$

where $f_{a}$ and $f_{b}$ are the area proportions for the maize strip and soybean strip, respectively; *k_a_* and *k_b_* are the extinction coefficients of maize and soybean, respectively; the extinction coefficients for maize and soybean were 0.43 and 0.64 [37], respectively; *Fia_black_* and *Fib_black_* are the view factors for beneath the first layer of the maize and soybean shape model, respectively; *Fiia_black_* and *Fiib_black_* are the view factors for beneath the second layer of maize and soybean shape models, respectively; *LAI_a_* and *LAI_b_* are the maize *LAI* and soybean *LAI*, respectively; and *LAI_ia_* and *LAI_iia_* are the *LAI* for the upper and lower layers of maize, respectively. The *Fia_black_*, *Fib_black_*, *Fiia_black_*, *Fiib_black_*, *LAI_ia_*, and *LAI_iia_* are as follows:

(12) $${\mathrm{Fiia}}_{\mathrm{black}}=\frac{\sqrt{{H_{b}}^{2}+W_{a}^{2}}-H_{b}}{W_{a}}$$

(13) $${\mathrm{Fiib}}_{\mathrm{black}}=\frac{\sqrt{{H_{b}}^{2}+W_{b}^{2}}-H_{b}}{W_{b}}$$

(14) $${\mathrm{Fia}}_{\mathrm{black}}=\frac{\sqrt{{\left(H_{a}-H_{b}\right)}^{2}+W_{a}^{2}}-(H_{a}-H_{b})}{W_{a}}$$

(15) $${\mathrm{Fib}}_{\mathrm{black}}=\frac{\sqrt{{\left(H_{a}-H_{b}\right)}^{2}+W_{b}^{2}}-(H_{a}-H_{b})}{W_{b}}$$

(16) $${\mathrm{LAI}}_{\mathrm{ia}}=\frac{H_{a}-H_{b}}{H_{a}}\times {\mathrm{LAI}}_{a}$$

(17) $${\mathrm{LAI}}_{\mathrm{iia}}=\frac{H_{b}}{H_{a}}\times {\mathrm{LAI}}_{a}$$

where *H_a_* and *H_b_* are the maize height and soybean height, respectively; *W_a_* and *W_b_* are the strip widths for the maize and soybean, respectively.

(18) $$F_{a}=F1+F2+F3+F4+F5+F9$$

(19) $$F_{b}=F6+F7+F8$$

where *F_a_* and *F_b_* are the fractions of PAR intercepted by maize, *F_a_*, and by soybean, *F_b_*.

The light-interception fraction of soybeans in a monocropping is calculated using the *LAI* and *k* value of the soybean monocropping [38,39], where the extinction coefficient is 0.34:

(20) $$F_{b}=1-e^{-k_{mon}{LAI}_{mon}}$$

### 2.5. Radiation Use Efficiency (RUE)

The formula for calculating the radiation use efficiency (*RUE*) (g MJ^−1^) is as follows [37,40]:

(21) $$\mathrm{RUE}=\frac{\mathrm{ADM}}{\sum \mathrm{IF}}$$

where *ADM* is the accumulated dry matter (g m^−2^), and *I* is the amount of incoming PAR per day (PAR, MJ m^−2^). The total daily solar radiation values were multiplied by 0.5 to convert the total radiation to PAR. *F* represents the fraction of (intercepted photosynthetically active radiation) PAR intercepted by the intercropped soybeans on a given day. Therefore, *F* was used to calculate the cumulative *IPAR* for intercropped soybeans.

### 2.6. Statistical Analysis

Data organization was performed using Excel 2021, and statistical analysis and visualization were performed using the R programming language. To separate mean differences among treatments at the 0.05 level, Duncan’s Multiple Range Test was used. Linear fitting was used to assess the effects of soybean canopy PAR on soybean morphology, dry matter accumulation, and yield at different growth stages.

## 3. Results

### 3.1. Accumulated PAR

**Figure 3:**
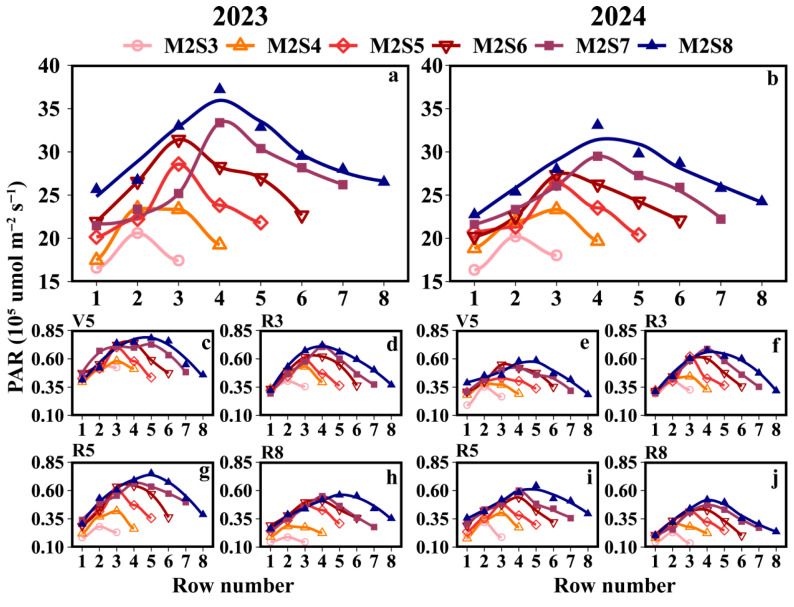
Accumulated PAR interception of the soybean canopy across different rows under different strip-width treatments in soybean–maize strip intercropping. (**a**,**b**) represents the accumulated PAR measured by the soybean canopy sensor from the V5 to R8 stages, and (**c**–**j**) represents the distribution of PAR within the soybean canopy during each growth stage. V5: five-leaf stage; R3: podding initiation stage; R5: grain filling stage; R8: maturity stage. The row number refers to those in Figure 2.

The accumulated PAR interception in soybean canopies across different strip widths in intercropping is shown in Figure 3a. Two years’ data show that all treatments in the strip intercropping system exhibited a single-peak pattern of daily PAR interception. PAR accumulation in intercropped soybeans was consistently higher in the western rows than that in the eastern rows. At the R3, R5, and R8 growth stages, the PAR accumulation in the western rows was 5.4%, 12.7%, and 10.5% higher than that in the eastern rows, respectively (Figure 3b). During the experimental period, the western rows showed a 6.5% increase in PAR accumulation compared to the eastern rows. The mean PAR accumulation values (×10^5^ μmol m^−2^ s^−1^) in the western rows across treatments followed the order M2S8 (27.1) > M2S7 (26.7) > M2S6 (24.0) > M2S5 (22.4) > M2S4 (19.5) > M2S3 (17.8), while those in the eastern rows were M2S8 (26.9) > M2S7 (23.5) > M2S6 (22.8) > M2S5 (21.1) > M2S4 (18.2) > M2S3 (16.5). The cumulative PAR under the canopy of solo soybeans refers to Supplementary Tables S1 and S3.

### 3.2. Daily PAR

**Figure 4:**
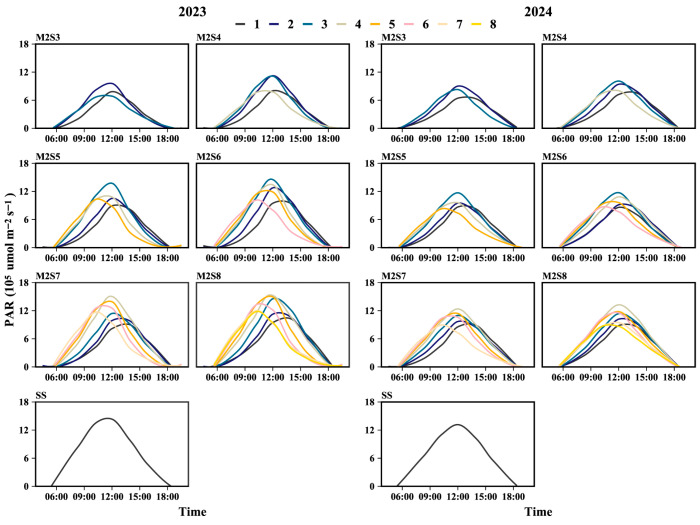
Daily PAR accumulation from V5 to R8 stages in soybean canopies across different rows in soybean–maize strip intercropping. V5: five-leaf stage, R3: podding initiation stage, R5: grain filling stage, R8: maturity stage. The row number refers to those in Figure 2.

The daily variation in PAR accumulation from the V5 to the R8 stages across soybean rows is shown in Figure 4. Two years’ data showed that the daily PAR in the soybean canopy under sole cropping peaked at 12:00, whereas for strip intercropping treatments, the time of the PAR peak relative to 12:00 varied with strip width. The western rows consistently reached their maximum PAR accumulation at 11:30, whereas the eastern rows peaked at 13:00. For treatments M2S3, M2S4, M2S5, M2S6, M2S7, and M2S8, the PAR peak time in the eastern rows was delayed by 45, 60, 45, 26, 35.5, and 18 min, respectively, compared with that at 12:00. Conversely, the PAR peaks in the western rows occurred 45, 52.5, 75, 86.2, 35, and 60 min earlier than 12:00, respectively.

### 3.3. RUE

**Figure 5:**
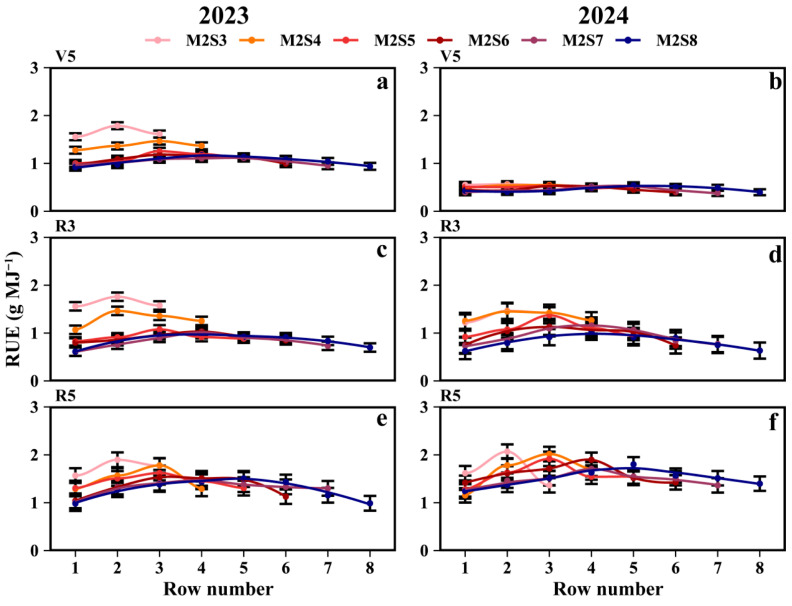
Radiation use efficiency (RUE) of soybean in different rows with different strip widths in soybean–maize strip intercropping. V5: RUE at the five-leaf stage (**a**,**b**), R3: RUE at the podding initiation stage (**c**,**d**), R5: RUE at the grain filling stage (**e**,**f**), R8: maturity stage. The row number refers to those in Figure 2. Black vertical lines indicate duncan at *p* = 0.05 (n = 3).

As shown in Figure 5, two-year data revealed that intercropped soybean RUE during the V5, R3, and R5 stages of the western rows increased by 3.8%, 5.1%, and 5.4%, respectively, compared to the eastern rows. At the R5 stage (Figure 5e,f), the RUE (g MJ^−1^) for the eastern rows across treatments from M2S3 to M2S8 was 1.59, 1.22, 1.42, 1.35, 1.33, and 1.30, respectively, while for the western rows it was 1.58, 1.49, 1.46, 1.39, 1.40, and 1.34. For the RUE of sole soybeans, refer to Tables S1 and S2.

### 3.4. Leaf Area

**Figure 6:**
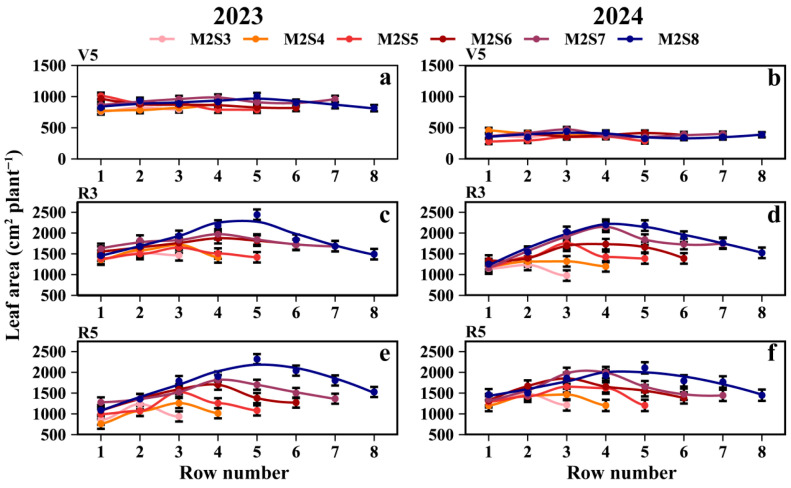
Leaf area of soybean across rows at different strip widths in soybean–maize strip intercropping. V5: Leaf area at the five-leaf stage (**a**,**b**), R3: Leaf area at the podding initiation stage (**c**,**d**), R5: Leaf area at the grain filling stage (**e**,**f**), R8: maturity stage. The row number refers to those in Figure 2. Black vertical lines indicate duncan at *p* = 0.05 (n = 3).

At the R5 stage, the mean leaf area per intercropped soybean plant (cm^2^ plant^−1^) for each treatment followed the order: SS (2218.9) > M2S8 (1731.9) > M2S7 (1563.5) > M2S6 (1485.6) > M2S5 (1312.6) > M2S4 (1177.3) > M2S3 (1173.4) (Figure 6e,f). The mean leaf area of the intercropped soybeans in the western rows was 6.7% higher than that in the eastern rows. From M2S3 to M2S8, the mean leaf areas (cm^2^ plant^−1^) of intercropped soybean in the eastern rows were 1086.2, 982.5, 1194.7, 1356.9, 1488.8, and 1511.1, respectively; those in the western rows were 1076.4, 1109.4, 1291.3, 1540.7, 1615.4, and 1731.6; and those in the middle rows were 1357.6, 1308.6, 1590.8, 1701.2, 1906.6, and 2063.5. For the leaf area of solo soybeans, refer to Tables S1 and S2.

### 3.5. Dry Matter

**Figure 7:**
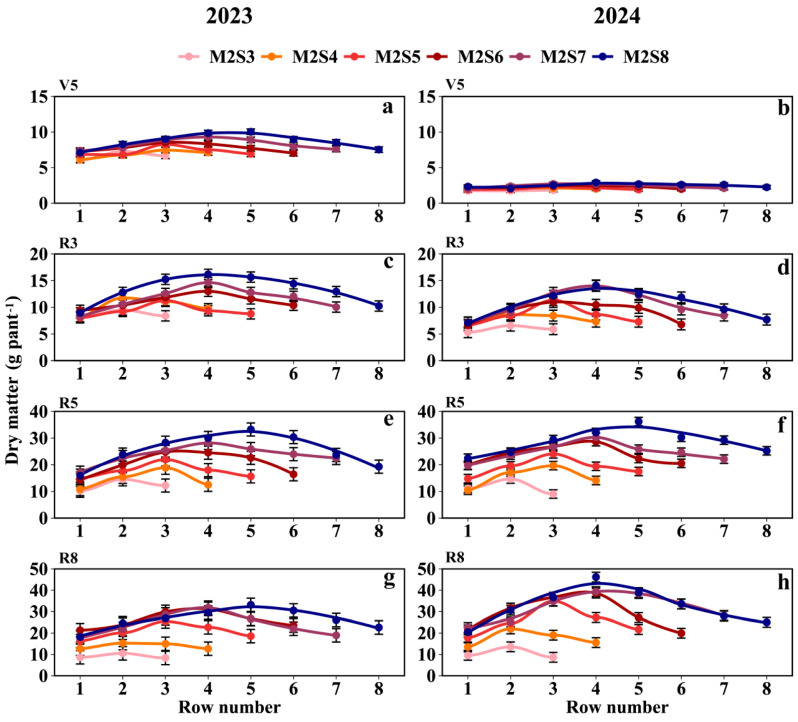
Dry matter weight of soybean across rows at different strip widths in intercropping. V5: Dry matter at the five-leaf stage (**a**,**b**), R3: Dry matter at the podding initiation stage (**c**,**d**), R5: Dry matter at the grain filling stage (**e**,**f**), R8: Dry matter at the maturity stage (**g**,**h**). The row number refers to those in Figure 2. Black vertical lines indicate duncan at *p* = 0.05 (n = 3).

As shown in Figure 7 and Tables S1 and S2, the dry matter of intercropped soybean in the western rows was greater than that in the eastern rows. At the R5 stage, the dry matter weight per plant (g plant^−1^) followed the order SS (52.9) > M2S8 (27.1) > M2S7 (24.2) > M2S6 (22.1) > M2S5 (18.4) > M2S4 (14.9) > M2S3 (11.9) (Figure 7e,f). From M2S3 to M2S8, the dry matter weight in the western rows was 7.8% higher than that in the eastern rows. The dry matter weights (g plant^−1^) for the eastern rows across treatments from M2S3 to M2S8 were 10.7, 10.6, 16.8, 19.6, 22.6, and 24.1, respectively; those in the western rows were 10.6, 13.3, 17.6, 20.5, 24.1, and 26.3; and those in the middle rows were 14.6, 17.8, 23.0, 26.2, 29.2, and 33.0, respectively.

### 3.6. Grain Yield

**Figure 8:**
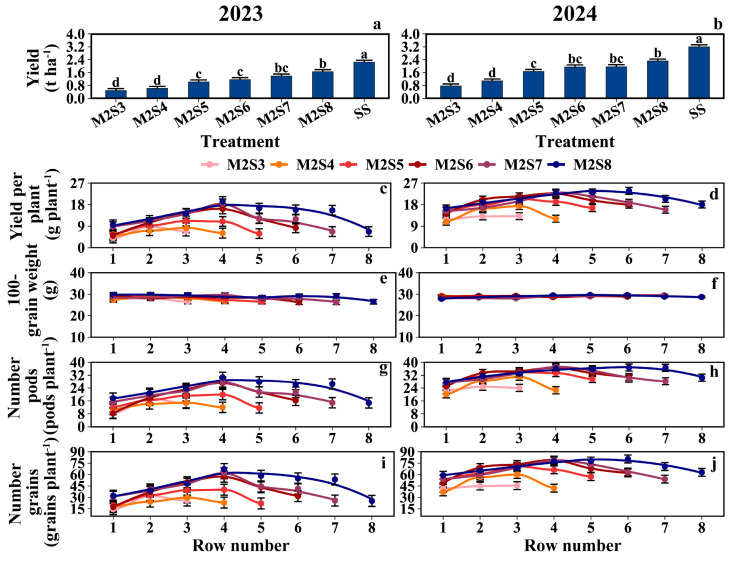
Distribution of soybean yield and yield composition across rows at different strip widths in soybean–maize strip intercropping. The row number refers to those in Figure 2. (**a**,**b**) Soybean yield per hectare in metric tons, (**c**,**d**) Average Yield per plant, (**e**,**f**) Weight of 100 soybeans, (**g**,**h**) Average number of pods per soybean plant, (**i**,**j**) Average number of grains per soybean plant. Black vertical lines indicate duncan at *p* = 0.05 (n = 3). Different lowercase letters indicate differences among treatments at *p* < 0.05.

As shown in Figure 8a,b, the grain yield per unit area (kg ha^−1^) increased with strip width. From M2S3 to M2S8, the yields (g plant^−1^) of the eastern rows were 8.0, 7.9, 12.1, 12.5, 14.3, and 15.1, respectively; the yields of the western rows were 10.1, 9.1, 13.2, 14.6, 14.5, and 16.7; and the yields of the middle rows were 11.0, 12.3, 15.8, 18.7, 20.7, and 20.5. The mean soybean yield in the western rows was 10.6% higher than that in the eastern rows (Figure 8c,d). Except for the 100-grain weight, the number of pods per plant, and the number of grains per plant, the row yield in the west row showed 12%, 11.4%, and 20.3% higher values than those in the east row, respectively (Figure 8e–j). For the yield composition of solo soybeans, refer to Tables S1 and S2.

### 3.7. Correlation Analysis

#### 3.7.1. PAR Versus Dry Matter, Leaf Area, and RUE

**Figure 9:**
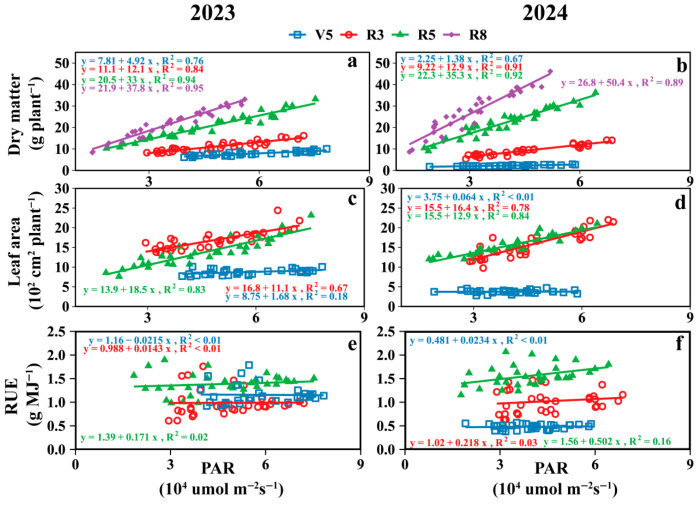
The relationship between soybean canopy PAR at different growth stages and dry matter (**a**,**b**), leaf area (**c**,**d**) and RUE (**e**,**f**) of intercropped soybean at key growth periods. V5: five-leaf stage; R3: podding initiation stage; R5: grain filling stage; R8: maturity stage.

PAR was positively correlated with the dry matter and leaf area of the intercropped soybeans, whereas it was negatively correlated with RUE (Figure 9 and Figure S2). The greatest effect of PAR on soybean dry matter occurred during the R8 stage with the highest slope, while for leaf area and RUE, it occurred during the R5 stages.

**Figure 10:**
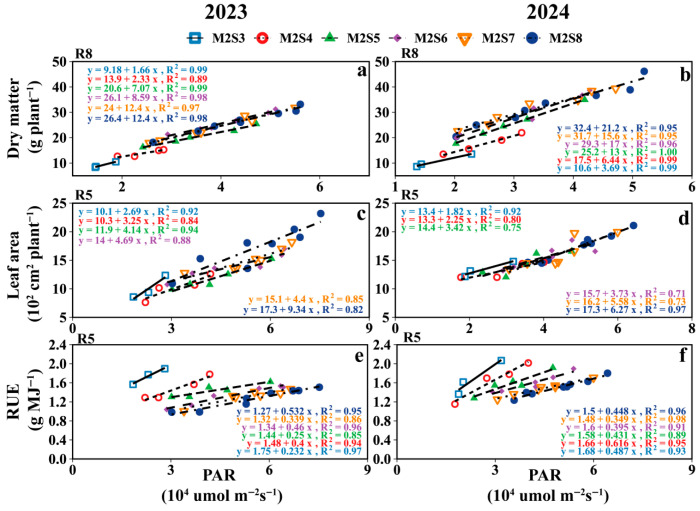
The relationship between soybean canopy PAR at different growth stages and dry matter (**a**,**b**), leaf area (**c**,**d**) and RUE (**e**,**f**) of intercropped soybeans under different strip widths. V5: five-leaf stage and soybean canopy PAR at V5; R3: podding initiation stage and soybean canopy PAR at R3; R5: grain filling stage; R8: maturity stage and soybean canopy PAR at R8.

As shown in Figure 10, the relationship between PAR and intercropped soybean dry matter/leaf area was characterized by the following: adding one additional soybean row to the intercropping strip increased dry matter and leaf area per intercropped soybean plant by 3.9 g and 111.7 cm^2^, respectively. Meanwhile, the greatest effect of PAR on RUE was observed in the M2S6 treatments. For other growth stages, refer to Figure S2 and Tables S1 and S2.

#### 3.7.2. PAR Versus Yield

**Figure 11:**
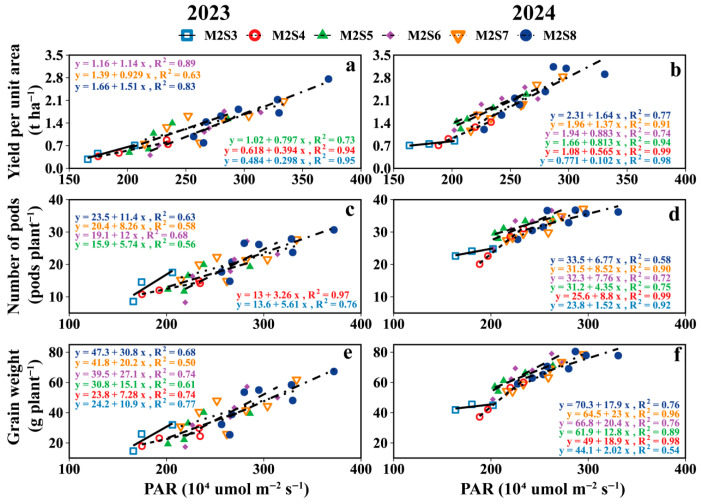
The relationship between soybean yield per unit area (**a**,**b**), Number pods per plant (**c**,**d**) and grain weight per plant (**e**,**f**) with cumulative PAR in the soybean canopy from the V5 to R8 stages.

As shown in Figure 11, the accumulated PAR interception of the intercropped soybean canopy in strip intercropping was positively correlated with yield per unit area, number of pods per plant, and grain weight per plant; the greatest effect of PAR on yield was observed in M2S8. When PAR increased 2.1 (×10^5^ μmol m^−2^ s^−1^) in the soybean canopy, yield increased 4.9 g plant^-1^ for each row of intercropped soybeans.

## 4. Discussion

### 4.1. Effect of Strip-Width on Inter-Row Heterogeneity of PAR in Soybean

Soybean–maize strip intercropping leads to spatial niche differentiation [26,29]. Soybean canopy light conditions are significantly affected by maize plant height. When maize plants are taller than soybeans, the combined effects of their relative heights and diurnal variations in solar elevation angle (diurnal inherently implies 12 h cycles) result in distinct direct sunlight exposure patterns: only western-side soybeans receive direct sunlight in the morning, soybeans across the entire strip at noon, and only eastern-side soybeans in the afternoon. This directly reduces soybean light interception (Figure 3 and Figure 4), which is consistent with the findings of Kou et al. [29,33]. Furthermore, this study further demonstrated that this light distribution pattern (induced by maize height and diurnal solar elevation changes) not only gives rise to differences in light interception between soybean rows (Figure 4) but also leads to discrepancies in accumulated PAR peaks among soybeans across different time periods. This variation in PAR, in turn, manifests as a distinct gradient within each strip width, which is closely correlated with the distance from the maize rows. Notably, our observations documented that the cumulative light interception in the western soybean rows exceeded that in the eastern rows. Potentially contributing to this disparity are site-specific variations in morning and evening sunlight exposure, as well as the orientation of the planting rows. Specifically, a clear positive relationship was observed: the greater the distance of soybean rows from maize rows, the higher the PAR accumulation in the soybean canopy. Accordingly, in soybean–maize strip intercropping systems, selecting shorter maize varieties is recommended to minimize the disparities in light exposure between soybean rows, thereby optimizing the overall light environment for intercropped soybeans.

### 4.2. Effect of Strip-Width on Inter-Row Heterogeneity of RUE in Soybean

Different strip-intercropping width configurations exhibit variations in RUE, which may have a limiting threshold. In this study, intercropping improved the light energy utilization rate of soybeans. However, Zhang et al. [41] found that the light energy utilization rates of intercropped wheat and cotton showed no significant difference compared with monocropping. In apricot–millet intercropping, millet exhibited higher light energy utilization efficiency than in monocropping [42]. Similarly, Liu et al. found that soybean light energy utilization rates increased when grown in strip intercropping with maize [7]. This study revealed that the RUE of soybean strongly responded to changes in strip width across treatments, particularly pronounced in the inner rows (Figure 5). With the increase in strip width, the east-row and west-row RUE in soybean strips gradually stabilized; when the strip width exceeded a certain width, the RUE between adjacent rows tended to stabilize and no longer exhibited significant variation (Figure 5). This conclusion is consistent with the findings of Kou et al. and Wang et al. in maize–soybean strip intercropping [29] and maize–peanut intercropping [11], where RUE increased with an increase in strip width ratio. Notably, RUE exhibited a gradient variation among the soybean rows, with soybeans in the middle rows exhibiting higher photosynthetic efficiency than those in the edge rows. The western rows exhibited a higher RUE than the eastern rows. The cause of this phenomenon may be that solar elevation and maize shading reduce the total solar radiation received by soybeans while increasing the proportion of diffuse light, thereby boosting the radiation use efficiency (RUE) of the intercropped soybeans [19,33,43], altering light quality (e.g., red: far-red ratio), and causing PAR increases to outpace RUE gains, thereby influencing productivity [19,44]. With planting density maintained constant in this experiment, expanding the strip width enhanced PAR accumulation into the intercropped soybean canopy. However, the soybean plant spacing concomitantly increased as strip widths widened. This adjustment in plant spacing may have further amplified intercropped soybean radiation use efficiency (RUE), leading to gradient changes in leaf area and dry matter accumulation. Additionally, soybeans growing under shade may partially offset the negative effects by regulating competition for water, nitrogen, and light [40,45]. These results underscore the finite benefits of increasing the strip width, which is a critical consideration for optimizing field configurations.

### 4.3. Effect of Strip-Width on Inter-Row Heterogeneity of Yield, Dry Matter, and Leaf Area in Soybean

In the intercropped soybean canopy, PAR gradients drove morphological differences, with leaf area and dry matter exhibiting distance-dependent gradient changes relative to the maize rows within each strip width (Figure 6 and Figure 7). Soybean is sensitive to light variations, as previously reported [46,47]. Our results also confirmed this phenomenon. Both traits (leaf area and dry matter) increased with strip width without apparent saturation, although their rate of increase first rose and then declined with wider strips. Notably, PAR most strongly affected the dry matter at the R8 stage, followed by the R5 stage, which was contrary to expectations, as the R5 (grain fill stage) should dominate accumulation. This pattern was validated by the correlations between PAR and intercropped soybean dry matter and leaf area (Figure 10). Intercropped soybean RUE increased under reduced light and elevated diffuse radiation [39,48,49], coupled with the morphological plasticity of intercropped soybeans (e.g., leaf area and height adjustments) [48,50]. Diurnal light intensity differences (morning vs. evening) mirrored east–west row disparities in dry matter and leaf area. The effects of strip width on morphological development and yield—common across maize/peanut [21], millet/mungbean [50], and soybean/maize [6] systems—were also evident here.

Increased strip width alters the light distribution in intercropped soybean canopies, thereby increasing the intercropped soybean leaf area, dry matter, and yield [13,51,52]; this phenomenon was attributed to the increased strip width and the plant spacing, which improved light distribution within the intercropped soybean population [53]. This was also confirmed in our study (Figure 10). During the single-day illumination, the PAR in the intercropped soybean canopy showed subtle differences, which were differences that were amplified throughout the reproductive period, leading to variations in yields and yield components of intercropped soybeans across different rows (Figure 8). The yield of intercropped soybean in each row exhibited a gradient change related to the distance from maize rows within each strip width. Meanwhile, the difference in PAR between the eastern and western sides of the intercropped soybean strip leads to the difference in soybean yield between the east and west sides. Therefore, appropriate field configurations for different climates will need to be considered in the future.

### 4.4. Quantifying Inter-Row Heterogeneity in Soybean–Maize Strip Intercropping

A critical revelation is the pronounced heterogeneity in light interception responses across intercropped soybean rows—responses that systematically varied with their positional distance from maize rows and strip width gradients. Intercropping systems exhibit three-dimensional optical properties [7,8,54], and soybean leaf area, dry matter, and yield show gradient variations. This phenomenon essentially stems from changes in the strip width, which alter the distribution ratio between scattered light and direct light, as corroborated by previous research [33,39,48,49]. Therefore, a model that simulates changes in the dynamic gradient of light in the intercropped soybean canopy based on the daily dynamics of light can be developed to improve the accuracy of intercropped yield predictions. Unlike the uniform decreases observed in narrow strips, the RUE of intercropped soybeans in middle rows stabilized at wider strip widths. This phenomenon may arise from the combined effects of reduced maize shading pressure and enhanced light diffusion as intercropping strip width increases, creating a more stable microclimate for photosynthesis. This spatiotemporal variability underscores the necessity of quantifying intra-strip light microgradients for accurate yield prediction.

The intercropping system is highly complex, with numerous factors influencing crop light capture, growth, and yield formation. Crop models can simulate key physiological and ecological processes, yet it is impractical to collect extensive data on all aspects of the system. For example, the APSIM model could simulate the light interception and yield formation for both crops in strip intercropping [32], but it does not distinguish between direct radiation and diffuse radiation, two components that may be changed by row configuration [11,21]. Munz et al. [33] reported that variations in strip width resulted in different ratios of direct/diffuse light transmission between rows, leading to inter-row differences, and set RUE to a fixed value to simulate biomass accumulation [55]. Certain complex physiological and ecological processes exist—those “black box” aspects we cannot fully understand. Machine learning, however, offers a relatively good auxiliary approach to estimate key physiological processes [56]. Nazmus et al. [57] investigated integrating time-series growth data across multiple scales (intraday and interday), genotypes, and growth conditions, thereby establishing associations between genotypes and phenotypes. Specifically, we can utilize the existing data through machine learning algorithms—such as Extreme Gradient Boosting (XGboost) and Short Long-Term Memory (LSTM)—to quickly and accurately summarize the dynamics of canopy PAR gradients. Subsequently, we can derive canopy PAR distribution from meteorological data and refine existing crop growth models: these refined models can simulate the relationship between strip width and crop growth while also accurately predicting the growth processes and subtle differences during yield formation of intercropped crops [58]. These datasets established in this study provide support for assessing PAR competition between maize and soybean in strip intercropping and lay a foundation for the future integration of crop models and machine learning to interpret and simulate the process of strip intercropping.

## 5. Conclusions

In this study, we quantified the spatiotemporal heterogeneity of intercropped soybean in light interception, leaf area, biomass, RUE, yield, and their relationship across rows under six strip widths; RUE, dry matter accumulation, and leaf area of soybean in the western rows were 4.0%, 7.4%, and 6.7% higher than that of the eastern rows. Soybeans in M2S8 showed the highest yield and light interception, while M2S3 showed the highest RUE. These findings advance our understanding of resource partitioning in strip intercropping systems and provide a theoretical basis for designing climate-adaptive intercropping systems, particularly in regions with distinct solar trajectories.

## Data Availability

The data that support the findings of this study are available from the corresponding author upon reasonable request.

## Supplementary Materials

The following supporting information can be downloaded at: https://www.mdpi.com/article/10.3390/plants15020182/s1. Figure S1. Measurement position of photosynthetically active radiation (PAR). Red dots are sensor placement locations, even rows with the average of the middle two rows as the middle row, odd rows with a single row in the middle as the middle row, small numbers on both sides as the east row, large numbers as the west row. Row number 1 is the first row in the east, and row number 8 is the eighth row in the east. Tables S1–S3. Supplemented with information on solo soybeans (Includes on cumulative PAR under soybean canopies, PAR under soybean canopies at various growth stages, RUE, leaf area, dry matter, and yield per unit area), indicators comparison of solo soybean and intercropping soybean with different strip widths in 2023 and 2024. Different lowercase letters indicate significant differences among treatments (*p* < 0.05). Figure S2. The relationship between canopy accumulation PAR and dry matter (a,b), leaf area (c,d) and RUE (e,f). Linear fitting of data from each treatment at each stage of reproduction with PAR. And Tables S4 and S5. Fitting formula and R^2^ of PAR to dry matter, leaf area, and RUE at key growth stages in Figure S2. Fitting equations and R^2^ values for PAR with dry matter, leaf area, and RUE at key growth stages in 2023 and 2024. For an explanation of the stages, shown in Figure 4.
