# Supplementary material for: Effects of Strip Width on Inter-Row Heterogeneity in Light Interception and Utilization of Intercropped Soybeans
Source: Plants (Basel). 2026 Jan 7;15(2):182. doi: 10.3390/plants15020182 (PMC12844913; doi:10.3390/plants15020182)
Supplement: Supplementary file 1 [file plants-15-00182-s001.zip › plants-4047214-supplementary.pdf]

Supplementary for

Effects of strip width on inter-row heterogeneity in light interception and utilization of intercropped soybeans

Yue Li [1,2], Yao Zhang [1,2], Jiamiao Shi [1,2], Ruizhe Zhang [1,2], Lisha Zhang [1,2], Yuan Yang [1,2], Haichang Li[1,2], Lihua Wang [1,2], Tianyu Yuan [1,2], Sirong Huang [1,2], Xiaochun Wang [1,2], Feng Yang [1,2], Taiwen Yong [1,2], Jiang Liu[3], Yanhong Yan[4], Wenyu Yang [1,2,*], Yushan Wu [1,2,*]

[1] *College of Agronomy, Sichuan Agricultural University, Chengdu 611130, China*

[2] *Sichuan Engineering Research Center for Crop Strip Intercropping System, Key Laboratory of Crop Eco-physiology and Farming System in Southwest, Chengdu 611130, China*

[3] *College of Life Science, Sichuan Agricultural University, Yaan 625014, PR China.*

[4] *College of Grassland Science and Technology, Sichuan Agricultural University, Chengdu 611130, PR China.*

** Corresponding authors.*

E-mail address: mssiyangwy@sicau.edu.cn (WYY) and yushan.wu@sicau.edu.cn(YSW)

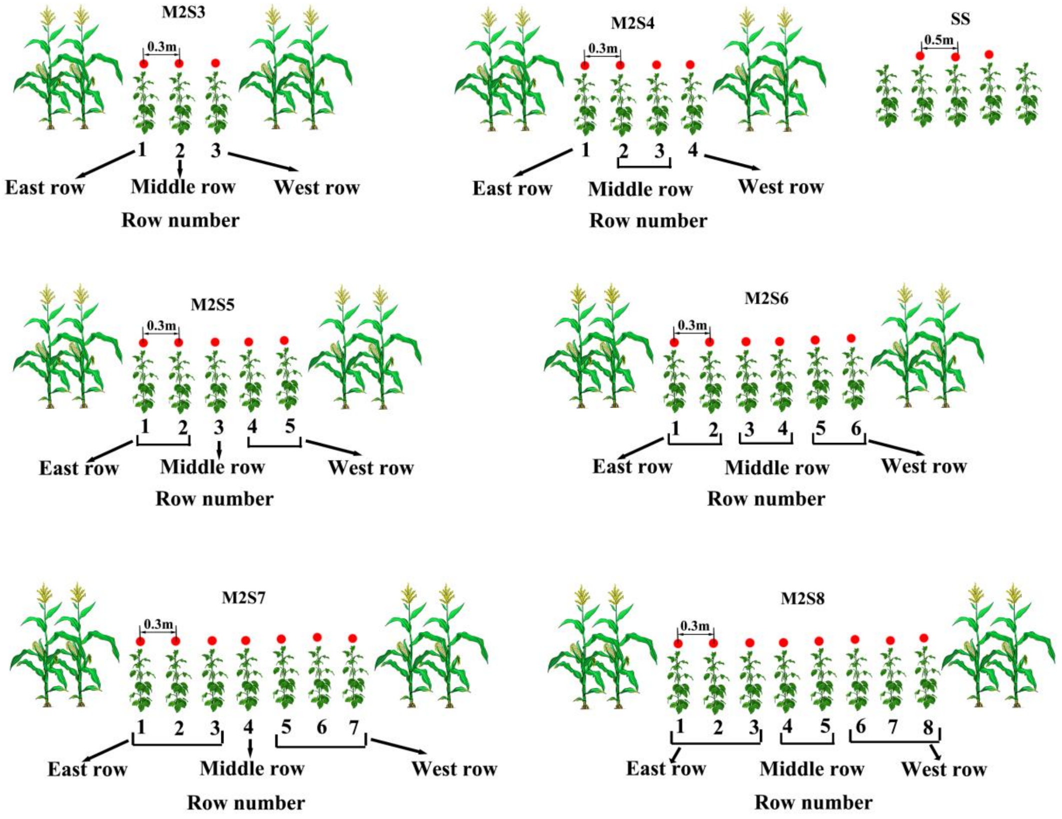

Figure S1 Measurement position of photosynthetically active radiation (PAR). Red dots are sensor placement locations, even rows with the average of the middle two rows as the middle row, odd rows with a single row in the middle as the middle row, small numbers on both sides as the east row, large numbers as the west row. Row number 1 is the first row in the east, and row number 8 is the eighth row in the east.

Table S1 Indicators comparison of monocrop soybean and intercropping soybean with different strip widths in 2023. Different lowercase letters indicate significant differences among treatments ($p < 0.05$). V5: five-leaf stage, R3: podding stage, R5: grain filling stage, R8: maturity stage.

Year	Growth stages	Treatment	Canopy PAR (×10^4μmol $m^{-2}s^{-1}$)	RUE (g MJ^{-1})	Leaf area (cm^2)	Dry matter (g)	F_b
2023	V5	M2S3	4.97	1.69±0.05a	793.1±30.1d	6.7±0.3e	0.38±0.01d
		M2S4	4.99	1.39±0.04b	812.2±26cd	6.9±0.2e	0.41±0.01d
		M2S5	5.31	1.14±0.04c	875.7±23.3bcd	7.3±0.2de	0.47±0.01c
		M2S6	5.93	1.1±0.03c	869.7±21.3bcd	7.8±0.2cd	0.49±0.01bc
		M2S7	6.28	1.05±0.03c	927.7±19.7b	8.3±0.2bc	0.51±0.01b
		M2S8	6.21	1.05±0.03c	893.2±18.4bc	8.7±0.2b	0.5±0.01b
		SS	9.49	0.77±0.05d	1021.3±52.1a	11.9±0.4a	0.62±0.01a
	R3	M2S3	3.64	1.83±0.06a	1453.3±77.9d	8.7±0.6e	0.29±0.01g
		M2S4	4.40	1.42±0.05b	1520.3±67.5cd	10.2±0.5cde	0.37±0.01f
		M2S5	4.38	0.99±0.05c	1490.6±60.4d	9.3±0.5de	0.44±0.01e
		M2S6	4.96	0.96±0.04c	1731.1±55.1bc	11.1±0.4cd	0.5±0.01d
		M2S7	4.97	0.88±0.04c	1783.3±51b	11.6±0.4c	0.53±0.01c
		M2S8	5.46	0.88±0.04c	1839.4±47.7b	13.3±0.4b	0.58±0.01b
		SS	8.13	0.71±0.06d	4009.5±134.9a	30.4±1.1a	0.97±0.01a
	R5	M2S3	2.32	1.96±0.1a	1010.5±81.6e	12.3±1.5f	0.29±0.02g
		M2S4	3.18	1.62±0.09b	1026.8±70.7e	14.4±1.3ef	0.35±0.01f
		M2S5	4.30	1.56±0.08bc	1186.4±63.2de	17.7±1.2de	0.4±0.01e
		M2S6	4.88	1.43±0.07bc	1397.6±57.7cd	20.4±1.1cd	0.47±0.01d
		M2S7	5.36	1.4±0.07bc	1507.7±53.4c	23.7±1bc	0.52±0.01c
		M2S8	5.59	1.34±0.06c	1729.3±50b	25.6±0.9b	0.56±0.01b
		SS	8.27	0.98±0.1d	2435.4±141.4a	55.5±2.7a	0.87±0.02a
	R8	M2S3	1.60			9.2±1.9d	
		M2S4	2.44			13.9±1.6d	
		M2S5	3.57			20.6±1.5c	
		M2S6	4.11			26.1±1.3b	
		M2S7	3.86			24±1.2bc	
		M2S8	4.39			26.4±1.1b	
		SS	6.30			57.8±3.2a	

Table S2 Indicators comparison of monocrop soybean and intercropping soybean with different strip widths in 2024. Different lowercase letters indicate significant differences among treatments ($p < 0.05$). V5: five-leaf stage, R3: podding stage, R5: grain filling stage, R8: maturity stage.

Year	Growth stages	Treatment	Canopy PAR (×10^4μmol $m^{-2}s^{-1}$)	RUE (g MJ^{-1})	Leaf area (cm^2)	Dry matter (g)	F
2024	V5	M2S3	2.70	0.56±0.03a	381±23.9ab	1.8±0.1c	0.31±0.02ab
		M2S4	3.32	0.53±0.03ab	408.7±20.7a	2.1±0.1abc	0.33±0.02a
		M2S5	3.77	0.51±0.03ab	314.2±18.5b	2±0.1bc	0.3±0.01ab
		M2S6	4.34	0.47±0.02bc	382.5±16.9ab	2.3±0.1ab	0.34±0.01a
		M2S7	4.24	0.45±0.02bc	392.3±15.7a	2.4±0.1ab	0.35±0.01a
		M2S8	4.54	0.46±0.02bc	373.1±14.7ab	2.5±0.1a	0.34±0.01a
		SS	6.50	0.41±0.03c	343.7±41.4ab	2.5±0.2a	0.28±0.02b
	R3	M2S3	3.40	1.41±0.1a	1118.8±83.1f	5.9±0.7d	0.28±0.01g
		M2S4	3.82	1.39±0.09a	1256.7±71.9ef	7.9±0.6c	0.34±0.01f
		M2S5	4.27	1.1±0.08b	1431.6±64.4de	8.5±0.5c	0.41±0.01e
		M2S6	4.65	0.98±0.07bc	1535.5±58.7cd	9.1±0.5bc	0.46±0.01d
		M2S7	4.84	0.95±0.06bc	1728.7±54.4bc	10.5±0.4b	0.51±0.01c
		M2S8	5.06	0.83±0.06c	1801.5±50.9b	10.6±0.4b	0.56±0.01b
		SS	7.16	0.53±0.1d	2699.3±143.9a	17.5±1.2a	0.91±0.01a
	R5	M2S3	2.36	1.75±0.1a	1336.3±84.3d	11.6±1.1f	0.31±0.01f
		M2S4	2.98	1.72±0.09a	1327.8±73d	15.3±0.9e	0.36±0.01e
		M2S5	3.49	1.63±0.08a	1438.7±65.3cd	19.1±0.8d	0.42±0.01d
		M2S6	4.12	1.64±0.07a	1573.7±59.6bcd	23.8±0.8c	0.48±0.01c
		M2S7	4.43	1.51±0.07a	1619.4±55.2bc	24.7±0.7c	0.51±0.01c
		M2S8	4.93	1.53±0.06a	1734.4±51.6b	28.7±0.7b	0.55±0.01b
		SS	6.85	0.97±0.1b	2002.4±146.1a	50.3±1.9a	0.84±0.01a
	R8	M2S3	1.70			10.6±1.7e	
		M2S4	2.49			17.5±1.5d	
		M2S5	2.95			25.2±1.3c	
		M2S6	3.20			29.3±1.2bc	
		M2S7	3.39			31.7±1.1b	
		M2S8	3.58			32.4±1.1b	
		SS	5.76			64.1±3a	

Table S3 Comparison of canopy accumulated PAR and yield of monocrop soybean and intercropping soybean with different strip widths. Different lowercase letters indicate significant differences among treatments ($p < 0.05$).

Year	Treatment	Canopy accumulated PAR （$\times 10^6$μmol $m^{-2}s^{-1}$）	Yield （t ha^{-1}）
2023	M2S3	1.82	0.5±0.1d
	M2S4	2.09	0.6±0.1d
	M2S5	2.33	1±0.1c
	M2S6	2.63	1.2±0.1c
	M2S7	2.69	1.4±0.1bc
	M2S8	2.99	1.7±0.1b
	SS	3.80	2.2±0.1a
2024	M2S3	1.82	0.8±0.1d
	M2S4	2.10	1.1±0.1d
	M2S5	2.25	1.7±0.1c
	M2S6	2.38	1.9±0.1bc
	M2S7	2.51	2±0.1bc
	M2S8	2.72	2.3±0.1b
	SS	3.50	3.2±0.1a

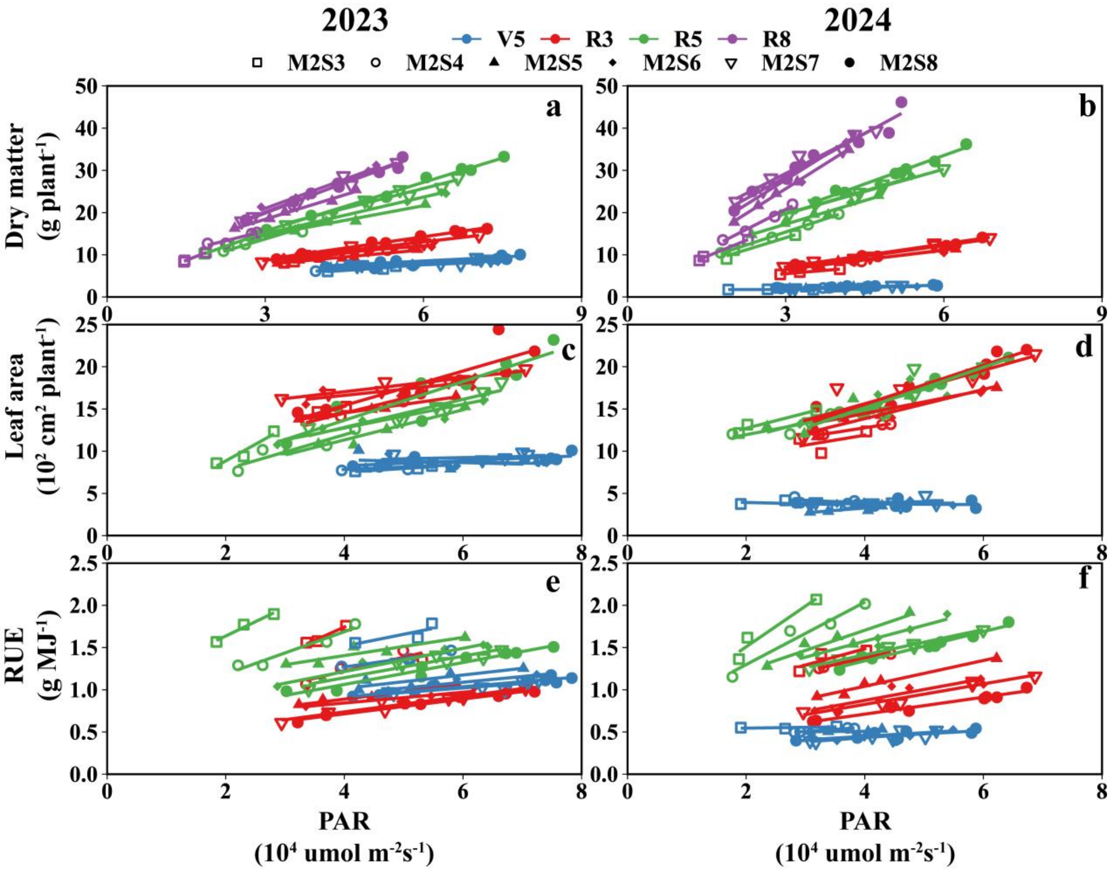

Figure S2 The relationship between canopy accumulation PAR and leaf area, dry matter and RUE. Linear fitting of data from each treatment at each stage of reproduction with PAR. V5: five-leaf stage, R3: podding stage, R5: grain filling stage, R8: maturity stage.

Table S4 Fitting formula and R^2 of PAR to dry matter, leaf area, and RUE at key growthstages in Figure S2. V5: five-leaf stage, R3: podding stage, R5: grain filling stage, R8: maturity stage.

Year		Treatment	V5		R3		R5		R8	
			Fitting formula	R^2	Fitting formula	R^2	Fitting formula	R^2	Fitting formula	R^2
2023	Dry matter	M2S3	y = 6.68 + 0.81x	0.87	y = 8.67 + 1.04x	0.99	y = 12.34 + 3.03x	1.00	y = 9.18 + 1.66x	0.99
		M2S4	y = 6.87 + 0.96x	0.94	y = 10.2 + 2.61x	0.89	y = 14.42 + 6x	0.97	y = 13.92 + 2.33x	0.90
		M2S5	y = 7.27 + 1.17x	0.89	y = 9.33 + 2.36x	0.95	y = 17.66 + 5.41x	0.97	y = 20.62 + 7.07x	0.99
		M2S6	y = 7.78 + 1.26x	0.93	y = 11.11 + 2.6x	0.83	y = 20.43 + 9.62x	0.98	y = 26.09 + 8.59x	0.98
		M2S7	y = 8.25 + 1.52x	0.67	y = 11.57 + 4.99x	0.94	y = 23.7 + 8.41x	0.95	y = 23.99 + 12.43x	0.97
		M2S8	y = 8.66 + 2.53x	0.86	y = 13.31 + 6.84x	0.99	y = 25.58 + 15.68x	0.99	y = 26.45 + 12.44x	0.98
	Leaf area	M2S3	y = 793.07 + 42.51x	0.90	y = 1453.26 + 105.54x	0.86	y = 1010.47 + 269.43x	0.92		
		M2S4	y = 812.22 + 38.77x	0.23	y = 1520.26 + 277.27x	0.95	y = 1026.77 + 325.35x	0.84		
		M2S5	y = 875.69 - 38.17x	0.04	y = 1490.63 + 206.99x	0.99	y = 1186.37 + 414.32x	0.94		
		M2S6	y = 869.68 - 26.19x	0.05	y = 1731.09 + 201.15x	0.61	y = 1397.58 + 468.95x	0.88		
		M2S7	y = 927.72 + 35.81x	0.12	y = 1783.35 + 280.47x	0.93	y = 1507.67 + 440.08x	0.85		
		M2S8	y = 893.17 + 119.63x	0.52	y = 1839.38 + 778.58x	0.75	y = 1729.32 + 934.33x	0.82		
	RUE	M2S3	y=1.69+0.14x	0.67	y=1.83+0.17x	0.96	y=1.96+0.25x	0.97		
		M2S4	y=1.39+0.14x	0.96	y=1.42+0.29x	0.81	y=1.62+0.43x	0.94		
		M2S5	y=1.14+0.18x	0.73	y=0.99+0.19x	0.93	y=1.56+0.27x	0.84		
		M2S6	y=1.1+0.18x	0.84	y=0.96+0.19x	0.85	y=1.43+0.49x	0.96		
		M2S7	y=1.05+0.14x	0.77	y=0.88+0.34x	0.91	y=1.4+0.36x	0.86		
		M2S8	y=1.05+0.24x	0.90	y=0.88+0.35x	0.97	y=1.34+0.56x	0.95		

Table S5 Fitting formula and R^2 of PAR to dry matter, leaf area, and RUE at key stages in Figure S2. V5: five-leaf stage, R3: podding stage, R5: grain filling stage, R8: maturity stage.

Year		Treatment	V5		R3		R5		R8	
			Fitting formula	R^2	Fitting formula	R^2	Fitting formula	R^2	Fitting formula	R^2
2024	Dry matter	M2S3	y = 1.77 - 0.02x	0.06	y = 5.94 + 0.86x	0.98	y = 11.6 + 3.87x	0.93	y = 10.59 + 3.69x	0.99
		M2S4	y = 2.11 + 0.07x	0.33	y = 7.9 + 1.29x	0.96	y = 15.34 + 6.88x	1.00	y = 17.52 + 6.44x	0.99
		M2S5	y = 2.04 + 0.27x	0.60	y = 8.47 + 3.6x	0.98	y = 19.07 + 6.73x	0.98	y = 25.23 + 12.96x	1.00
		M2S6	y = 2.27 + 0.37x	0.80	y = 9.06 + 3.92x	0.89	y = 23.77 + 7.56x	0.93	y = 29.28 + 16.98x	0.96
		M2S7	y = 2.39 + 0.61x	0.87	y = 10.48 + 6.16x	0.98	y = 24.72 + 8.25x	0.98	y = 31.73 + 15.57x	0.95
		M2S8	y = 2.46 + 0.66x	0.63	y = 10.6 + 6.42x	0.98	y = 28.72 + 11.66x	0.98	y = 32.42 + 21.15x	0.95
	Leaf area	M2S3	y = 381.02 - 18.21x	0.15	y = 1118.8 + 95.27x	0.27	y = 1336.31 + 181.65x	0.92		
		M2S4	y = 408.73 - 30.43x	0.25	y = 1256.7 + 120.33x	1.00	y = 1327.82 + 224.51x	0.80		
		M2S5	y = 314.19 + 59.45x	0.65	y = 1431.62 + 403.03x	0.94	y = 1438.73 + 342.27x	0.75		
		M2S6	y = 382.51 + 3.67x	0.01	y = 1535.45 + 363.59x	0.84	y = 1573.71 + 372.78x	0.71		
		M2S7	y = 392.31 + 25.27x	0.07	y = 1728.7 + 673x	0.78	y = 1619.38 + 557.63x	0.73		
		M2S8	y = 373.1 - 10.78x	0.01	y = 1801.49 + 859.54x	0.91	y = 1734.4 + 626.93x	0.97		
	RUE	M2S3	y=0.56+0.01x	0.35	y=1.41+0.16x	0.71	y=1.75+0.51x	0.93		
		M2S4	y=0.53+0.05x	0.96	y=1.39+0.18x	0.96	y=1.72+0.64x	0.94		
		M2S5	y=0.51+0.01x	0.02	y=1.1+0.36x	0.97	y=1.63+0.44x	0.88		
		M2S6	y=0.47+0.09x	0.65	y=0.98+0.35x	0.82	y=1.64+0.4x	0.91		
		M2S7	y=0.45+0.1x	0.58	y=0.95+0.42x	0.95	y=1.51+0.35x	0.97		
		M2S8	y=0.46+0.13x	0.62	y=0.83+0.38x	0.96	y=1.53+0.46x	0.96		
